# The blood pressure control and arteriosclerotic cardiovascular risk among Chinese community hypertensive patients

**DOI:** 10.1038/s41598-021-98745-8

**Published:** 2021-09-24

**Authors:** Shijun Liu, Hanyan Yuan, Caixia Jiang, Jue Xu, Xin Qiu, Jun Luo

**Affiliations:** 1 Department of Chronic and Non-Infection Disease Control and Prevention, Hangzhou Center for Disease Control and Prevention, Mingshi Road No.568, Hangzhou, 310021 China; 2 Gongshu District Center for Disease Control and Prevention, Hangzhou, China

**Keywords:** Epidemiology, Cardiovascular diseases, Hypertension

## Abstract

The present study aimed to describe the blood pressure (BP) control rate and 10-years arteriosclerotic cardiovascular disease (ASCVD) risk estimation among community hypertensive patients. A total of 196,803 subjects were enrolled. The control rates calculated as the intensive (SBP < 130 mmHg and DBP < 80 mmHg) and standard (SBP < 140 mmHg and DBP < 90 mmHg) threshold. Multivariable logistic analysis was employed to assess the associations between cardiovascular factors and BP control. Sensitivity, specificity and Youden’s index were used to identify the ability of high risk of ASCVD estimation by different thresholds. The control rate was 16.34% and 50.25% by the intensive and standard threshold, respectively. Besides regular medication, the risk factors for BP control included older age, male, unhealthy lifestyle, obesity, dyslipidemia and abnormal FPG. 25.08% of subjects had high risk of 10-years ASCVD estimation. The sensitivity, specificity and Youden’s index of intensive threshold was 84.37%, 16.15% and 0.51%, and were significantly different from 50.55%, 50.42% and 0.98% of the standard threshold, respectively. Half of community hypertensive patients did not control BP, and nearly a quarter have high risk of 10-years ASCVD risk estimation. The intensive threshold resulted in a one-third reduction in the control rate compared to the standard threshold. No matter which threshold was used, a single BP control status seemed not a suitable indicator for identification of high risk of 10-years ASCVD risk estimation.

## Introduction

Hypertension is prevalent in China. The survey conducted from 2012 to 2015 indicated that 23.2% of the Chinese adult population ≥ 18 years of age had hypertension, with the treatment and control rates of 50% and 20%^1^. Uncontrolled hypertension has been estimated at 750,000 deaths by cardiovascular disease (CVD) every year^2^. Therefore, Chinese government had issued a strategy to provide free Essential Public Health Service (EPHS) in 2009. As one of the content, the general practitioners (GPs) in the primary health care system (named community health care center, CHC) provided health management for hypertensive patients, includes regular follow-up and intervention, medication service, physical examination, etc.^3^ Since then, the blood pressure (BP) control rate of general population had achieved certain results according to a comparison of cross-section surveys, rising from 6.1% in 2002, 13.8% in 2012 to 16.8% in 2015^1^. Studies have shown that the management of hypertension by GPs was feasible and efficient in China, a significant reduction in the level of BP could be found by implementing a comprehension intervention^3–5^. However, these studies did not report the status of modifiable cardiovascular factors closely linked to the management of hypertension as lifestyle, obesity, lipid and glucose levels, etc.

The 2017 American Heart Association/American College of Cardiology (AHA/ACC) guidelines changed the diagnostic threshold and the management goal of BP from systolic BP (SBP) < 140 mmHg and diastolic BP (DBP) < 90 mmHg (standard threshold) to SBP < 130 mmHg and DBP < 80 mmHg (intensive threshold)^6^. This shift was based on the significant hazard ratios for coronary heart disease (CHD) and stroke of the comparison between SBP of 130 to 139 mmHg or DBP of 85 to 89 mmHg and SBP < 120 mmHg and DBP < 80 mmHg, will significantly increase the number of uncontrolled patients^7^. According to the 2018 Chinese Guidelines for Prevention and Treatment of Hypertension^8^, SBP of 130 to 139 mmHg and DBP of 80 to 89 mmHg was classified as high normal BP level, and SBP < 130 mmHg and DBP < 80 mmHg was an alternative goal of treatment if patients can tolerant the intensive treatment. Several studies towards different population concluded that the intensive goal of the management of hypertension would be benefit for cardiovascular events prevention^7,9–11^. However, a Chinese research suggested that drug treatment was not cost-effective compared with non-drug treatment for high normal BP (stage I hypertension in AHA/ACC) aged ≥ 65 years without cardiovascular disease in China^12^. We noted that ≥ 10% of 10-year atherosclerotic cardiovascular disease (ASCVD) estimation was the important trigger in use of intensive threshold for treatment^6^.

Comprehensive assessment and intervention of modifiable risk factors was an important component of hypertension management. Consequently, the present study aims to describe the distribution of control rates and cardiovascular factors by different thresholds among hypertensive patients managed by GPs, and to display their 10-year ASCVD risk estimation. Different from the previous observation among general population, this data was collected from a real-world and the subjects were hypertensive patients who attended the governmental EPHS project.

## Results

### General characteristics of subjects

A total of 196,803 individuals included in this analysis, 44.18% of males and 55.82% of females, with an average age of 69.31 ± 8.73 years. Majority of subjects (89.12%) were elderly patients aged 60 years and above. The average value of SBP and DBP were 141.29 ± 18.19 mmHg and 80.18 ± 10.00 mmHg. Overall, the medication rate of subjects was 90.71%, 88.71% of regular medication and 2.00% of intermittent medication. 34.67% and 16.39% of subjects was current smoking and drinking in males, in contrasted with 0.95% and 1.83% in females. 19.50% of subjects exercise every day, 9.17% of them combined with CVD. The distribution of variables was significantly different between male and female groups (Table 1).

**Table 1:** General characteristics of subjects.

Characteristics	Category	Total	Male	Female	P value
Number of subjects (N/%)		196,803 (100.00)	86,945 (44.18)	109,858 (55.82)	
Age (years)		69.31 ± 8.73	70.01 ± 8.44	68.76 ± 8.92	< 0.001
Age groups (%)	< 60 years	10.88	6.86	14.06	< 0.001
	≥ 60 years	89.12	91.14	85.94	
BMI (kg/m^2^)		24.29 ± 3.22	24.21 ± 3.36	24.36 ± 3.38	< 0.001
WC (cm)		83.91 ± 8.30	85.38 ± 8.04	82.75 ± 8.33	< 0.001
SBP (mmHg)		141.29 ± 18.19	140.27 ± 17.64	142.10 ± 18.57	< 0.001
DBP (mmHg)		80.18 ± 10.00	80.73 ± 10.03	79.75 ± 9.95	< 0.001
TC (mmol/L)		4.89 ± 1.05	4.69 ± 1.02	5.06 ± 1.05	< 0.001
TG (mmol/L)		1.68 ± 1.15	1.57 ± 1.16	1.76 ± 1.13	< 0.001
LDL (mmol/L)		2.57 ± 0.93	2.46 ± 0.88	2.66 ± 0.95	< 0.001
HDL (mmol/L)		1.54 ± 0.57	1.50 ± 0.56	1.57 ± 0.58	< 0.001
FPG (mmol/L)		5.58 ± 0.98	5.57 ± 0.99	5.59 ± 0.97	< 0.001
CRE (umol/L)		78.28 ± 24.08	87.26 ± 24.79	71.18 ± 20.94	< 0.001
Current smoke (%)		15.40	33.67	0.95	< 0.001
Current drink (%)	Daily	8.26	16.39	1.83	< 0.001
	Usual	6.39	12.62	1.46	
	None	85.34	70.99	96.71	
Exercise (%)	Daily	19.50	20.21	18.94	< 0.001
	Usual	16.36	16.45	16.28	
	None	64.14	63.34	64.77	
Medication adherence (%)	Regular	88.71	88.96	88.52	< 0.001
	Intermittent	2.00	1.84	2.13	
	Unused	9.29	9.20	9.36	
Combined with CVD (%)	No	90.83	89.52	91.87	< 0.001
	Yes	9.17	10.48	8.13	

### The BP control rate by intensive and standard threshold

**Figure 1:**
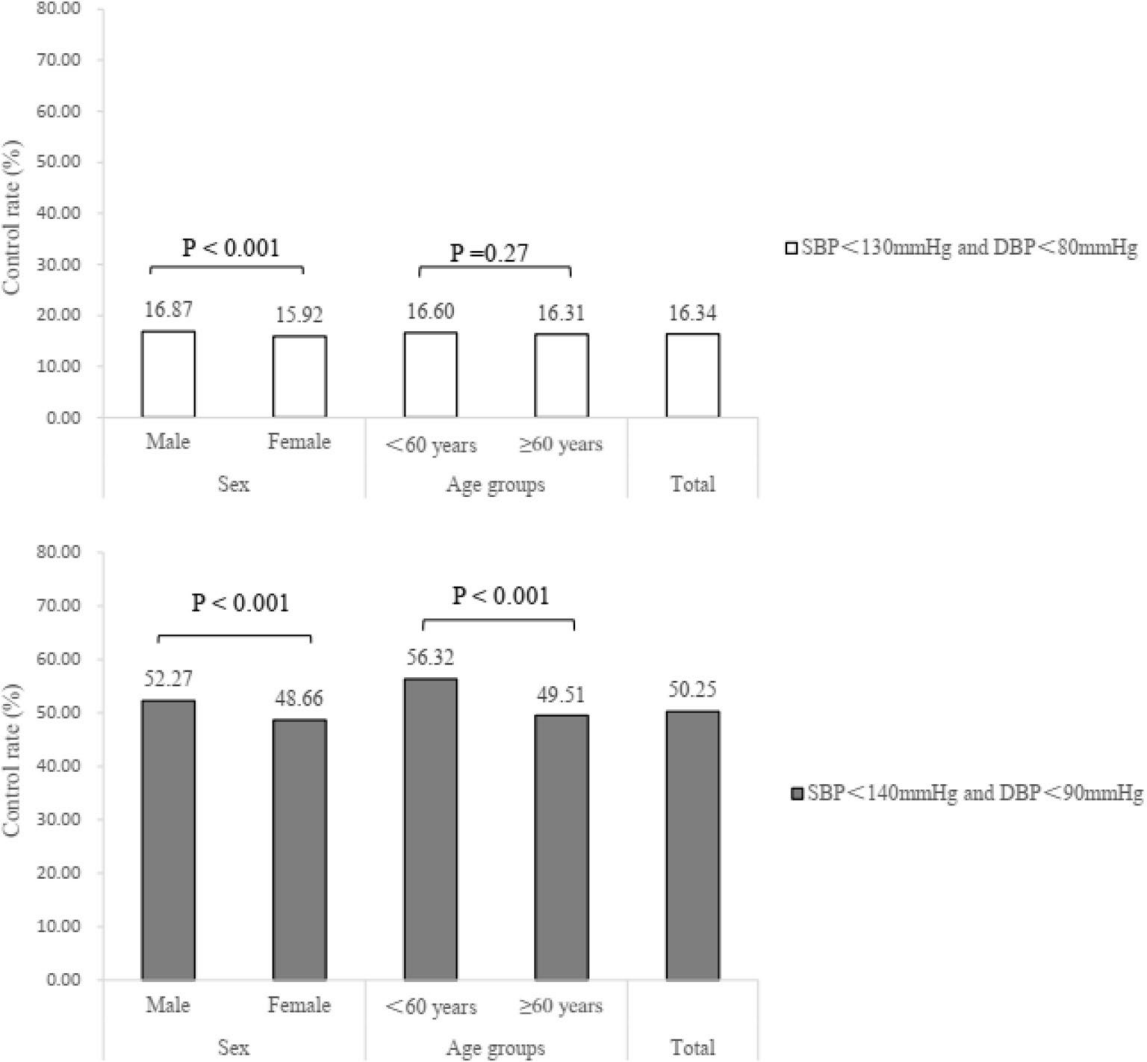
The BP control rate by different BP control thresholds.

The overall BP control rates were 16.34% and 50.25% by intensive and standard threshold, respectively (Fig. 1). Significance of control rate was found between male and female group, regardless of the BP thresholds (P < 0.001). Compared to the age of < 60 years group, the BP control rate was lower in the ≥ 60 years group only when using the standard thresholds (P < 0.001).

### Condition of BP control affected by cardiovascular factors, combination of CVD and medication adherence

Regardless of BP control thresholds, the distribution of unhealthy lifestyles, obesity, dyslipidemia, abnormal FPG, elevated CRE, CVD combination and medication adherence was significant between controlled and uncontrolled groups (Table 2). Unhealthy lifestyles was associated with poor BP control, with the highest OR (95% CI) 1.37 (1.26–1.48) of intensive threshold and 1.53 (1.45–1.62) of standard threshold when individuals had 3 items. Compared to those without elevated lipid elements, individuals with 1 items had OR (95% CI) 1.10 (1.07–1.34) by intensive threshold and 1.34 (1.18–1.51) by standard threshold, respectively. An increased trend of OR (95% CI) along with number of lipid items was found in both of thresholds (P_trend_ < 0.01). 19.58% of individuals already had abnormal FPG and 6.00% of them met the diagnostic criteria for type 2 diabetes. The OR (95% CI) was 1.36 (1.31–1.42) and 1.44 (1.36–1.52) by intensive threshold, 1.30 (1.26–1.33) and 1.49 (1.43–1.55) by standard threshold. Depending on level of CRE, 2.36% of individuals might suffer from impaired renal function, 3.91% of individuals had microalbuminuria. The significant relationship between elevated CRE and BP control was found when used standard threshold. Regular medication was a protective factor to BP control. The proportion of combined CVD was 9.17% and presented an inverse correlation with BP control.

**Table 2:** The proportion and hazard of cardiovascular factors, combined CVD and medication adherence.

Characteristics	Total	Intensive threshold		OR (95% CI)	Standard threshold		P value
		Uncontrolled	Controlled		Uncontrolled	Controlled	
**Unhealthy lifestyles (%)**							
0 item	15.94	15.40	18.69	1	14.04	17.82	1
1 item	67.21	67.76	64.39	1.28 (1.24–1.32)	69.22	65.21	1.35 (1.31–1.38)
2 items	13.80	13.75	14.05	1.24 (1.18–1.30)	16.60	14.00	1.38 (1.33–1.43)
3 items	3.05	3.09	2.87	1.37 (1.26–1.48)	3.14	2.97	1.53 (1.45–1.62)
**Overall obesity (%)**							
BMI < 24 kg/m^2^	48.41	47.48	53.15	1	46.79	50.02	1
24.00 ≤ BMI < 28 kg/m^2^	39.38	39.82	37.16	1.20 (1.17–1.23)	39.77	39.01	1.12 (1.10–1.14)
BMI ≥ 28 kg/m^2^	12.21	12.7	9.69	1.46 (1.40–1.52)	13.45	10.98	1.34 (1.30–1.38)
**Abdominal obesity (%)**							
WC < 90 cm (male), < 85c m (female)	66.31	65.35	71.20	1.00	63.39	69.20	1
WC ≥ 90 cm (male), ≥ 85 cm (female)	33.69	34.65	28.80	1.30 (1.27–1.34)	36.31	30.80	1.28 (1.26–1.31)
**TC (%)**							
< 6.2 mmol/L	90.33	89.92	92.45	1	89.11	91.55	1
≥ 6.2 mmol/L	9.67	10.08	7.55	1.36 (1.30–1.42)	10.89	8.45	1.30 (1.27–1.34)
**TG (%)**							
< 2.40 mmol/L	94.48	94.25	95.67	1	83.41	85.94	1
≥ 2.40 mmol/L	5.52	5.75	4.33	1.25 (1.21–1.30)	16.59	14.06	1.23 (1.20–1.26)
**LDL (%)**							
< 4.1 mmol/L	94.33	94.11	95.55	1	93.63	95.33	1
≥ 4.1 mmol/L	5.67	5.89	4.45	1.33 (1.26–1.41)	6.37	4.67	1.38 (1.32–1.43)
**HDL (%)**							
≥ 1.0 mmol/L	91.06	91.38	89.42	1	91.87	90.26	1
< 1.0 mmol/L	8.94	8.62	10.58	0.80 (0.77–0.84)	8.13	9.74	0.83 (0.81–0.86)
**Dyslipidemia (%)**							
0 item	70.38	69.90	82.80	1	68.94	71.80	1
1 item	20.94	21.14	19.92	1.10 (1.07–1.34)	21.43	20.46	1.10 (1.07–1.12)
2 items	7.58	7.81	6.41	1.26 (1.20–1.32)	8.36	6.71	1.28 (1.24–1.32)
≥ 3 items	1.10	1.14	0.88	1.34 (1.18–1.51)	1.27	0.93	1.41 (1.29–1.53)
**FPG (%)**							
< 6.1 mmol/L	80.42	79.63	84.43	1.00	78.04	82.79	1
6.1 ≤ FPG < 7.0) mmol/L	13.58	14.09	10.95	1.36 (1.31–1.42)	14.95	12.21	1.30 (1.26–1.33)
≥ 7.0 mmol/L	6.00	6.27	4.61	1.44 (1.36–1.52)	7.01	5.00	1.49 (1.43–1.55)
**CRE (%)**							
< 115 umol/L (male), < 107 umol/L (female)	93.73	93.83	93.22	1	93.35	94.11	1
115 ≤ CRE < 133 umol/L (male), 107 ≤ CRE < 124 umol/L (female)	3.91	3.83	4.31	0.90 (0.85–1.00)	4.03	3.78	1.08 (1.03–1.13)
≥ 133 umol/L (male), ≥ 124 umol/L (female)	2.36	2.34	2.47	0.97 (0.90–1.05)	2.62	2.11	1.23 (1.16–1.30)
**Combined with CVD (%)**							
No	90.83	90.99	90.00	1.00	91.11	90.55	1
Yes	9.17	9.01	10.00	0.90 (0.87–0.94)	8.89	9.45	0.91 (0.89–0.94)
**Medication adherence (%)**							
Regular	88.71	88.56	89.47	1.00	87.69	89.72	1
Intermittent	2.00	2.08	1.57	1.34 (1.22–1.48)	2.42	1.58	1.55 (1.45–1.65)
Unused	9.29	9.35	9.96	1.06 (1.01–1.10)	9.89	8.70	1.16 (1.13–1.20)

Note, age and sex was considered as adjusted variables in Logistic analysis.

### The distribution of 10-year ASCVD risk estimation

**Figure 2:**
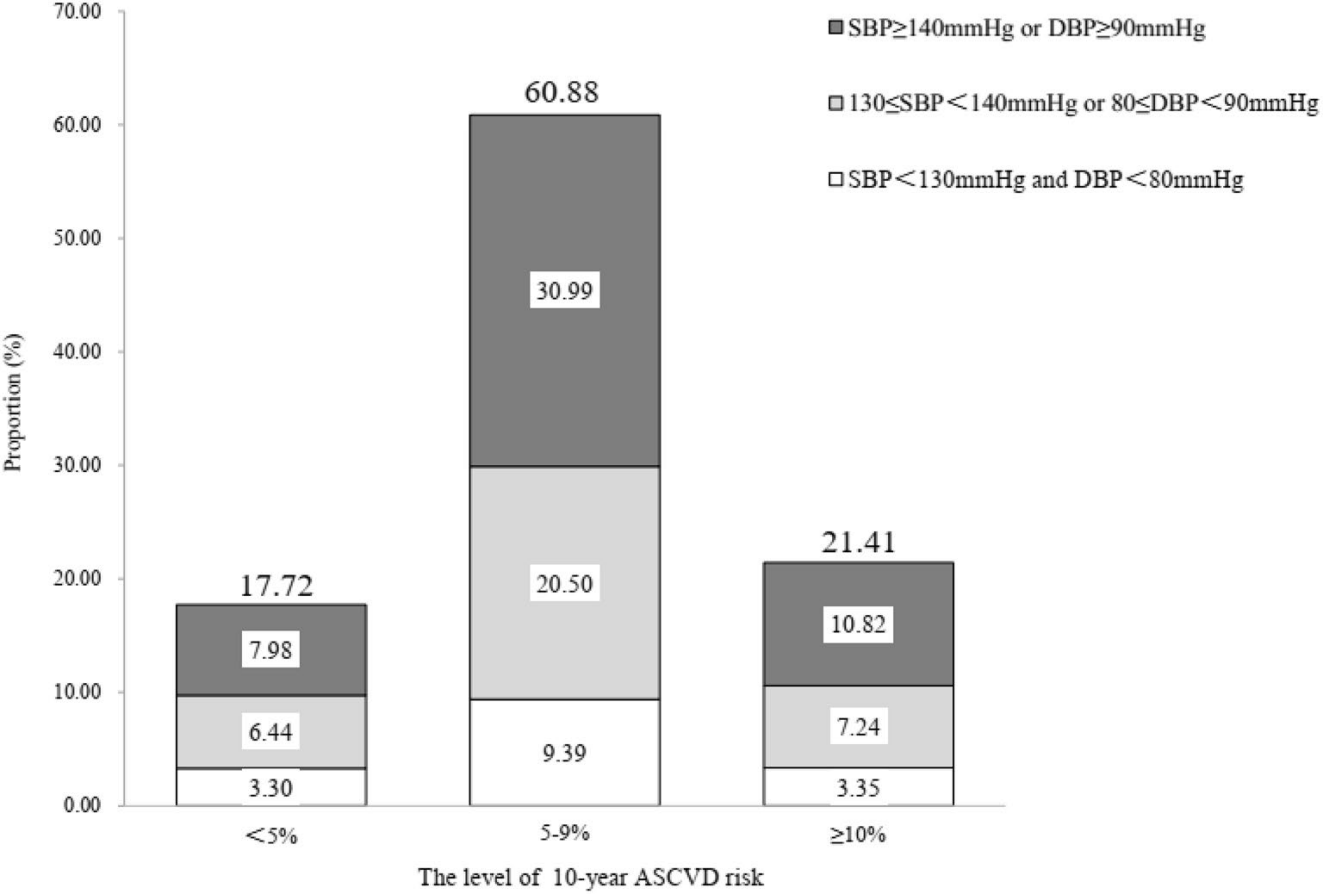
The 10-year ASCVD risk estimation by BP level.

**Figure 3:**
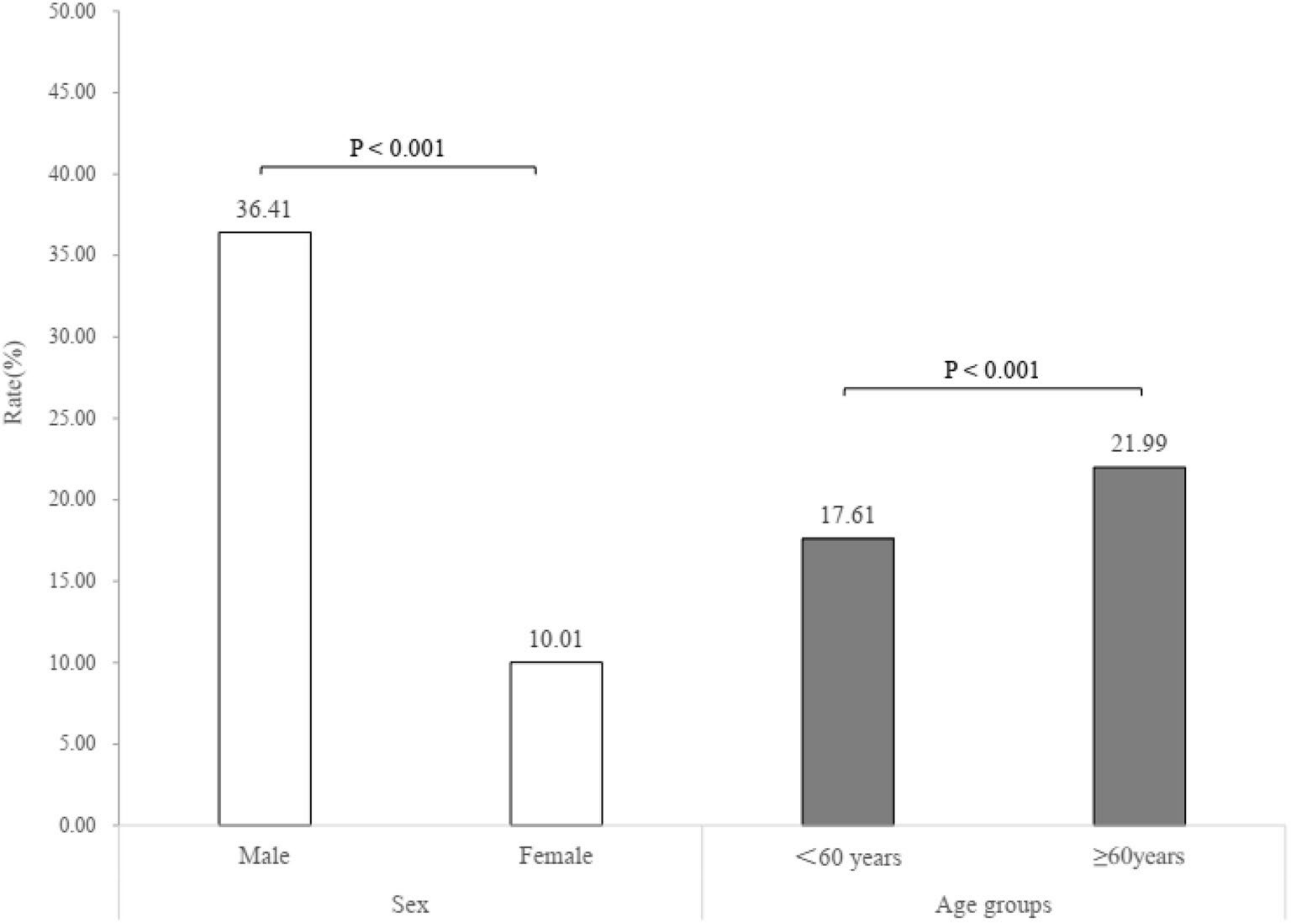
The high risk (≥ 10%) of 10-year ASCVD risk estimation by sex and age groups.

After excluding those had CVD or ≥ 80 years of age, a total of 154,992 individuals were enrolled into the analysis of 10-year ASCVD risk estimation. The proportion of low, medium and high risk of the 10-year ASCVD estimation was 21.41%, 60.88% and 17.72%, respectively. 10.82% of individuals with high risk (≥ 10%) could identify by standard threshold, and an extra 7.24% of individuals would consider as high risk by intensive threshold (Fig. 2). Men (36.41%) and individuals aged 60 years (21.99%) and above had higher risk of 10-year risk estimation, compared to women (10.01%) and individuals aged less than 60 years (17.61%) (Fig. 3). The Sen and Spe of intensive threshold were 84.37% and 16.15%, was significantly different from 50.55 and 50.42% of standard threshold. The YI value was 0.51% of intensive threshold and 0.98% of the standard threshold (Table 3).

**Table 3:** The sensitivity, specificity and Youden’s index towards 10-years ASCVD risk estimation (≥ 10%) by different thresholds.

	Intensive threshold	Standard threshold	P value
Sen (%)	84.37	50.55	< 0.001
Spe (%)	16.15	50.42	< 0.001
YI (%)	0.51	0.98	–

## Discussion

The present study reported a 50.25% of BP control rate by the standard threshold, and decreased to less than one third (16.34%) by the intensive threshold among community hypertensive patients managed by GPs in China. Unhealthy lifestyle, obesity, dyslipidemia and elevated FPG were risk factors for BP control, and regular medication was a protective factor for BP control. Part of subjects already had impaired renal function and suspected type 2 diabetes. 21.41% of individuals had high risk of 10-years ASCVD risk estimation. But a single BP control status was not a suitable indicator for this risk identification because of the insufficient YI of two thresholds. Subjects were female or aged less than 60 years had higher BP control rate and lower 10-years ASCVD risk estimation.

The China Hypertension Survey reported that 23.2% of Chinese adults with age of ≥ 18 years had hypertension, 40.7% of them used antihypertensive medications, and 15.3% had controlled BP by standard threshold^1^. To treated hypertension patients, the control rate ranged from 32.7 to 40.3% among different age groups^1^. Later, another multicenter project throughout 31 provinces in mainland China showed a higher prevalence, a lower treatment and controlled rate of hypertension among Chinese population ranged 35 to 75 years of age^13^. Subjects in the present study were different from participants of these two surveys, had attended in EPHS and could be provided with standard professional health management by GPs in CHC. Remarkably, the control rate of the subjects in this study was better than that of the same medication patients found in the general population, indicating that the EPHS project is beneficial to the BP control among hypertensive patients. Cluster randomized controlled trial had shown that trained governmental community health workers who were connected with existing public health care infrastructure led to a larger decrease in BP than usual care among adults with high BP, even in regions where the economy and society are relatively backward^14^. Data from 12 high-income countries showed that the control rate in treated patients could be up to 70% or higher, as in Canada, United States and Germany^15^. In contrast, only 10.3% of patients with hypertension achieved controlled BP because the medication rate was as low as 29.9% in low- and middle-income countries^16^. According to the speculation of published literature, the shift of BP threshold to SBP < 130 mmHg and DBP < 80 mmHg will significantly increase the number of hypertension or uncontrolled hypertension to about 50% of Asian adults^7^. Used the intensive threshold in the presented study, only achieved less than one third of control rate of the standard threshold. This result could be linked to the fact that the Chinese Hypertension Guidelines have always focused on the primary treatment goal (SBP < 140 mmHg and DBP < 90 mmHg)^17^.

Besides focused on the BP levels, the management of cardiovascular factors is equally important to hypertensive patients. Compared to those without or less of cardiovascular factors, hypertensive patients with more abnormal items were more difficult to control BP, regardless of the thresholds and age groups in the present study. These undoubted correlations had already announced by previous guidelines^6,8^. It should be noted that the prevalence of cardiovascular risk factors, especially as regards lifestyle and obesity, was reported in this study compared to the results of the general population. Among adult hypertension in American from 1999 to 2012, 15.5% were current smokers, 49.5% were obesity (BMI ≥ 28 kg/m^2^), and 63.2% had hypercholesterolemia, 27.2% had DM, and 15.8% had chronic kidney disease^18^. In the Chinese national survey (2012 to 2015) referred to above, the overall rate of obesity (≥ 28 kg/m^2^), current smoking and current drinking was 15.7%, 19.6% and 24.1% among sampled general population, respectively^13^. Based on the China Health and Retirement Longitudinal Study (2011–2012), the overall prevalence of heavy drinking, obesity, current smoking, and physical inactivity among middle-aged and older adults was 7.23%, 11.53%, 27.46% and 44.06%, respectively^19^. Compared with these outcomes, the prevalence of cardiovascular factors of the subjects in this survey did not obviously reflect the advantages of obtaining nonpharmacological management by GPs.

Diabetes mellitus and high blood pressure are often associated with each other because of both associating with increased insulin resistance^20^. In the present study, the BP control rates were lower for the abnormal FPG patients, compared with the normal FPG patients. Previous studies demonstrated that FPG in the pre-diabetes range significantly associated with future hypertension^21–23^. Simultaneously, controlling hypertension could affect hyperinsulinemia and be beneficial in preventing diabetes mellitus^24^. Additionally, individuals with confirmed T2DM were excluded from the observation. But some of the subjects already had abnormal FPG and a small number of them met the diagnostic criteria for T2DM. Implies that at least close to the physical examination time, some people may have cardiovascular risk factors that have not been taken seriously. The level of CRE was a marker for renal function in the hypertensive patients, and was a very potent independent risk factor for mortality, according to the research about prognostic value of CRE and effect of treatment of hypertension on renal function^25^. However, the present study found that the significant relationship between elevated CRE and BP control occurred when the standard threshold was used instead of intensive threshold. Systematic reviews suggested that intensive BP control did not provide additional benefit for renal outcomes compared with standard treatment^26,27^. Combined with CVD presented an inverse relationship with BP control, might be explained that individuals with CVD will have a higher risk assessment and cause more frequent intervention by GPs compared with those without CVD according to the EHPS.

The Chinese national survey showed about 9.5% of adults ranged 35 to 75 years had a high risk in future CVD, the proportion of 45 to 70 years old ranges from 12.6 to 17.7% and hypertension was the most prevalent risk factor among individuals with high risk^28^. In contrast, the gender and age distribution of high risk were similar, but obtained a higher proportion among subjects in the present study. The increased proportion could explain that subjects of the present study were hypertensive patients. Considering the ASCVD risk assessment was a trigger for implement of drug treatment under the intensive threshold, the present study used the high risk (≥ 10%) as the event to distinguish the identified ability of the two thresholds. Compared to the standard threshold, the intensive threshold with high sensitivity could additionally identify more high risk individuals but presented a low specificity. Significantly high value of YI index was observed for the standard threshold, but the value of two thresholds was insufficient. The guidelines demonstrated presence of multiple cardiovascular risk factors in individuals with hypertension results in high absolute risks for CHD and stroke, and persuaded antihypertensive drug treatment based on overall ASCVD risk assessment combined with BP levels may prevent more CVD events than treatment based on BP levels alone^6,8^. During the era of the first proposal, plenty of studies among varied population and regions showed newly defined stage I hypertension with high normal BP were associated with increased risk of incident CVD^9–11^. However, the SPRINT program reported those with lower baseline CVD risk had more harm than benefit from intensive treatment, whereas those with higher risk had more benefit^29^. The Chinese study pointed that stage I hypertension on cardiovascular risk is evidenced in young and middle-aged Chinese adults, but not in those age ≥ 60 years^30^. The latest system review compared the reduction in mortality and morbidity between lower (SBP < 135 mmHg and DBP < 85 mmHg) and standard BP targets (SBP of 140 to 160 and DBP of 90 to 100 mmHg) in the treatment of people with hypertension and a history of CVD, concluded that probably little to no difference in total mortality and cardiovascular mortality^31^. The evidence of this study presented that if the goal is to identify high risk ASCVD population, a single consideration of BP level could not provide reasonable benefits, and it seemed that there was no need to emphasize the lower target among the hypertension patients managed by GPs. In addition, in the context that the BP control was not sufficient even when the standard threshold was used, and emphasized a lower BP threshold need consider the concern including cost-effective, adverse events and compliance^12,32,33^.

The present study had several potential limitations. This study did not use representative sampling because the data was collected from the real-world, unavoidably existed defects such as biases of age and gender. An older average age of enrolled subjects might result in underestimating of BP control rates, compared to the overall community hypertensive patients managed by GPs. In contrast, a higher proportion of female subjects might cause an overestimation of BP control rates. However, it reasonable supposed that this regional data could demonstrate the current status of hypertensive patients under the national EPHS policy in China, because this service had unified technical specifications and strong governmental support. Factors as salt intake, family history, especially the medication regimen were not included in the analysis, could not provide more comprehensive information about the status of hypertension management and ASCVD estimation among this population. This study was a cross-sectional study and could not explain the causal relationship between factors and outcomes and more studies are still needed to confirm these findings and reach consensus.

## Subjects and methods

### Data source

In the 2017 national EPHS implementation and effectiveness evaluation, as the capital of Zhejiang Province, Hangzhou got the highest rank among 63 sampled cities around mainland in China. We reasonably consider that Hangzhou could be a sample of representing economically developed cities in China, to demonstrate the current status of comprehension prevention and treatment of hypertension health management in CHC.

The EPHS program in China is a national financial support project, proposed and approved by the National Health Commission. According to the EPHS, confirmed hypertensive patients aged at least 35 years who agreed to enroll hypertension health management, will be provided informed consent and established an electronic health management record, and receive comprehensive interventions by a work team combined with GPs, nurses and public health doctors in CHC. Unified electronic form which is designed by an authorized software company, assign to all CHCs in Hangzhou for collecting information. According to the national published specification, all staffs practicing EPHS had received a standard and unified training in information collection, diagnosis and treatment, operation in measurement and laboratory, also updated their skill and knowledge regularly by the official organization. All methods of this study were performed in accordance with the relevant guidelines and regulations. In list of comprehensive interventions, excepting of medicines, measurements as height, weight, blood pressure and body non-medical treatments as advice on diet, exercise, and mentality are provided for free to all patients. In contrast, laboratory testing as lipid, glucose is only free to individuals aged more than 60 years. Might due to the payment, part of patients would miss the advised examination, although the GPs had provided professional consultation.

### Subjects

The present study enrolled hypertensive patients who accepted health management and follow-up by GPs in CHC. Subjects who combined with confirmed diabetes were excluded for the difference in treatment schedule, the management goal of blood pressure and potential clinical hazards. Details as follows, 619,490 managed hypertension patients were entered in the electronic system in 2017. Individuals who combined with diabetes and did not have physical examination or necessary collected information were ejected. In all, a total of 196,803 individuals completed physical examination in 2017 were included in the present analysis. Compared to those not included in this study, the included individuals were older and had a higher proportion of females. As the above explanation, the population aged 60 years and above had free physical examination by EPHS program, reasonably caused the bias of the observed individuals. All subjects gave informed consent and had no disabilities or mental health problems.

### Variables and criteria

The cross-sectional data using to analysis included body measurements as height, weight, waist circumference and blood pressure, and laboratory testing as total cholesterol (TC), triglycerides (TG), low-density lipoprotein (LDL), high-density lipoprotein (HDL), fast plasma glucose (FPG) and serum creatinine (CRE). Above information was collected through physical examination. The analysis factors also included history of cardiovascular disease (CVD), medication adherence and lifestyle as smoking, drinking and physical exercise. This information was collected through follow-up by GPs.

The diagnostic criteria for hypertension was SBP ≥ 140 or DBP ≥ 90 mmHg (1 mmHg = 0.133 kpa) or taking antihypertensive drugs within two weeks. The BP control was calculated as the standard thresholds (SBP < 140 mmHg and DBP < 90 mmHg) and intensive threshold (SBP < 130 mmHg and DBP < 80 mmHg), respectively^6,8^. Subjects were asked to rest for at least 5 min, and then measure their BP at least twice and record the mean of the values. The BP measurement followed the key points, quiet and relaxed, standardized location and accurate readings. Definitions of cardiovascular risk factors were followed by 2018 Chinese Guidelines for Prevention and Treatment of Hypertension^8^ (Table 4). Smoking status is a dichotomous variable, and 1 cigarette per day at least in the past 6 months considered as current smoking. Using times per week to define the frequency of drinking and exercise status, ≥ 5 times per week, 1 to 4 times per week and less than 1 times per week defined as ‘daily’, ‘usual’ and ‘none’, respectively. Fasting status means no caloric intake at least 8 h.

**Table 4:** Risk factors influencing cardiovascular prognosis in hypertensive patients.

Cardiovascular risk factors	Definitions
Unhealthy lifestyle	Current smoking (yes), current drinking (everyday) or exercise < 4 times per week
Obesity	Body mass index (BMI) ≥ 28 kg/m^2^
Abdominal obesity	WC ≥ 90 cm (male), ≥ 85 cm (female)
Dyslipidemia	TC ≥ 6.2 mmol/L, TG ≥ 1.7 mmol/L, LDL ≥ 4.1 mmol/L or HDL < 1.0 mmol/L
Abnormal FPG	6.1 ≤ FPG < 7.0 mmol/L, FPG ≥ 7.0 mmol/L
Abnornal CRE	Slight increased CRE: 115–133 umol/L (male)
	Slight increased CRE: 107–124 umol/L (female)
	Elevated CRE: ≥ 133 umol/L (male)
	Elevated CRE: ≥ 124 umol/L (female)

### The 10-year ASCVD risk estimation

The 10-year ASCVD risk estimation was carried out according to 2016 Chinese guidelines for the management of dyslipidemia in adults^34^. A complete risk assessment as 21 combinations by the level of LDL, TC, FPG and hypertension with the number of other ASCVD risk factors, and was identified as low risk (< 5%), medium risk (5–9%) and high risk (≥ 10%). The current study listed only the risk assessment correlated to hypertension and used ≥ 10% to identify the high risk of ASCVD. Individuals with CVD and aged 80 years and above were excluded from this risk analysis, because these populations already had a high risk of 10-year risk of ASCVD^6^.

### Statistical analysis

Variables were summarized using means with standard deviation for continuous data, frequencies, percentages and proportions for categorical data. Independent t-tests and chi-squared tests were used to compare continuous and categorical variables, respectively. Logistic regression was used to analyze relationship between the condition of BP control and cardiovascular factors, combined CVD and medication adherence. Comparison of applicability between standard and intensive threshold was used the high risk (≥ 10%) of 10-year ASCVD risk as the event. Evaluation indicators included Sensitivity (Sen), Specificity (Spe) and Youden’s index (YI) were calculated as follows (Table 5). The significance of Sen and Spe between these two thresholds was conducted according to published literature^35^. P < 0.05 was the threshold for statistical significance. Analysis was performed using R version 3.4.0 (R Core Team, 2017. R: A language and environment for statistical computing. R Foundation for Statistical Computing, Vienna, Austria. URL https://www.R-project.org/).

**Table 5:** The calculated formula of sensitivity, specificity and Youden’s index.

The BP control by different thresholds	The 10-year ASCVD risk	
	≥ 10%	< 10%
Uncontrolled	a	b
Controlled	c	d
${\text{Sen}} = \frac{a}{{\left( {a + c} \right)}} \times 100\%$, ${\text{Spe}} = \frac{d}{{\left( {b + d} \right)}} \times 100\%$, YI = Sen + Spe − 1		
